# Development and validation of the psoriasis scale among the system of quality of life instruments for chronic diseases QLICD-PS (V2.0)

**DOI:** 10.1186/s12955-022-01970-6

**Published:** 2022-04-22

**Authors:** Qiongling Liu, Li Feng, Chonghua Wan, Jianfeng Tan, Jianbin Yu, Li Wang

**Affiliations:** 1 School of Nursing, Guangdong Medical University, Dongguan, 523808 China; 2 School of Health and Nursing, Wuchang University of Technology, Wuhan, 430223 China; 3 School of Humanities and Management, Research Center for Quality of Life and Applied Psychology, Key Laboratory for Quality of Life and Psychological Assessment and Intervention, Guangdong Medical University, Dongguan, 523808 China; 4 Department of Dermatology, The First Affiliated Hospital of Zhengzhou University, Zhengzhou, China

**Keywords:** Psoriasis, Quality of life, The disease-specific module, The general module, Psychometric properties

## Abstract

**Background and purpose:**

Psoriasis (PS) is difficult to cure with a high incidence. Therefore, the quality of life (QOL) of people with Psoriasis has caused widespread concern. Universal scales respond poorly to subtle changes caused by specific diseases, which makes it challenging to fully understand the impact of QOL in patients with psoriasis. In view of the deficiencies of the universal scale and the lack of a specific scale suitable for Chinese cultural background, this study aims to develop the psoriasis scale among the system of QOL instruments for chronic diseases QLICD-PS (V2.0).

**Methods:**

The scale QLICD-PS (V2.0) was developed based on the procedural decision-making approach and the experience of establishing scales at home and abroad. 122 patients with psoriasis were participated in measuring QOL 3 times before and after treatments. The reliability was assessed by test–retest reliability (Pearson’s correlation coefficients) and also internal consistency (Cronbach’s alpha coefficients). Qualitative analysis was adopted to evaluate content validity; item-domain correlation analysis, multi-dimensional scaling analysis, and factor analysis were adopted to evaluate the construct validity; the SF-36 scale was used as the criterion to evaluate the criterion-related validity due to lack of gold standard. Paired *t* tests were performed to evaluate the responsiveness on each domain/facet as well as the total of the scale, with Standardized Response Mean (SRM) being calculated.

**Results:**

The QLICD-PS was composed of the general module including 3 domains (28 items) and the psoriasis specific module (13 items). The Cronbach's α of the specific module, the general module and the total scale of the QLICD-PS was 0.78, 0.87 and 0.74 respectively, the split-half reliability of the specific module, the general module and the total scale was 0.81, 0.91 and 0.81, respectively, both indicating high reliability. Correlation and factor analysis confirmed good construct validity and criterion-related validity. After treatments, the score changes in the total scale were statistically significant with SRM being 0.5, showing moderate responsiveness.

**Conclusion:**

As the first psoriasis-specific QOL scale developed by the modular approach in Chinese, the QLICD-PS showed good reliability, validity and responsiveness, and could be used to measure the QOL of Patients with psoriasis specifically and sufficiently.

## Background

Psoriasis (PS) is a common chronic inflammatory immune-mediated skin disease caused by the interaction of genetic and environmental factors, etc. The incidence varies from regions and populations, and is related to factors such as race, geographic location, and environment [1]. Foreign surveys showed the incidence is 2% [2]. The Chinese epidemiological survey in 1984 showed that the total prevalence rate was 0.123%, with the prevalence rate in the north being higher than that in the south [3]. Due to the acceleration of China's urbanization, environmental pollution, intensified social competition and other factors, the current prevalence rate has increased to 0.47% [4]. In recent years, studies have shown that psoriasis is mainly related to infection, heredity, immune dysfunction, and endocrine disorders [5]. The disease has the characteristics of repeated attacks, skin damage, and long-lasting itching. It is a typical chronic psychosomatic disease, which has different degrees of impact on quality of life (QOL) of patients [6, 7].

With the transformation of the biomedical model to the bio-psycho-social model, the purpose of medicine was not simply to extend the survival of patients; the QOL had received more and more attention [8]. In the medical field, extensive QOL research began in the late 1970s with the purpose of exploring the influence of disease conditions and therapeutic interventions of patients, and thus the concept of health-related quality of life (HRQOL) was proposed [8, 9]. The term of HRQOL is often used to indicate QOL from the perspective of health care or medical services people experience. The WHO QOL Study Group defines QOL (in fact HRQOL) as individuals’ experience of life conditions related to their life goals, expectations, standards, and concerns in different cultures and value systems [10]. Generally speaking, QOL in medical field usually imply to HRQOL for short. Hence, the term of QOL and HRQOL are interchangeable in this study.

Psoriasis could systematic affect patients' health on various aspects, and thus QOL is needed. The purpose of the treatment of psoriasis was not only to relieve the symptoms of the patient's disease, but more importantly, to help the patient's psychological adaptation and improve the QOL. Therefore, to explore the QOL of patients with psoriasis and related influencing factors became an important problem in the prevention and treatment of psoriasis.

Since the 1980s, there had been an increasing number of researches on the QOL of patients with psoriasis. At first stage, some generic instruments such as SF-36 [11, 12], WHOQOL-100 [13, 14] were widely used for evaluating QOL of psoriasis. And then some dermatology scale were commonly used to assessment QOL of psoriasis including skin index (skindex) [15, 16], dermatology QOL index (DLQI) [17, 18], Dermatology QOL Scale(DQOLS) [19]. However, the specificity and sensitivity of these instruments were not strong enough when used for specific disease for they do not capture the symptoms and side effects specific to psoriasis.

Consequently, several disease-specific QOL measures for psoriasis have been developed including Psoriasis Disability Index (PDI) [20, 21], the psoriasis life stress inventory (PLSI) [22, 23], Salfore Psoriasis Index (SPI) [24], Psoriasis QOL Questionnaire 12 (PQOL-12) [25, 26], Psoriasis QOL Index (PSORQOL) [27], Psoriasis Stigma Experience Questionnaire (FSQ) [28] and so on (see Table 1 in detail).

**Table 1:** Some common quality of life scales for psoriasis

Quality of Life Scale	Country	Publication Year	Number of items	Domain
Skin index (Skindex)	USA	1997	29	Cognitive effects, social effects, physical limitations, physical disorders, depression, fear, embarrassment, anger
Dermatology life quality index (DLQI)	UK	1994	10	Physiology, psychology, daily activities, dressing, social entertainment, sports, work and study, family, sexual life, treatment
The Dermatology Quality of Life Scale (DQOLS)	UK	1997	41	Psychosocial health, physical activity, symptoms
Psoriasis Disability Index (PDl)	UK	1987	15	Daily activities; Study and work; Social interaction; Leisure; treatment
The psoriasis life stress inventory (PLSI)	USA	1995	15	Pressure to look good; Social stigma; Symptoms of disease or stress associated with treatment
Psoriasis quality of life questionnaire-12 (PQOL-12)	USA	2003	12	Psoriasis symptoms and the impact on patients' quality of life; The front and back of a simple figure of the body for patients to mark their affected skin; The patient's joint involvement and degree
Psoriasis index of quality of life (PSORQOL)	UK	2003	25	Investigate the patient's perceived self-image to others
Feeling of stigmatization of psoriasis (FSQ)	USA	1989	33	Fear of rejection; To feel defective; Sensitivity to the attitudes of others; Guilt and shyness; A positive attitude; The hidden illness

However, no scale for psoriasis has been developed based on the modular approach (a general/core module plus specific modules). Moreover, no Chinese version of any of these instruments is available for use in psoriasis patients in China. Compared with foreign countries, the development of Psoriasis scale in China started late. Considering cultural dependence of QOL, it was necessary to develop suitable psoriasis assessment scale under Chinese cultural background. Consequently, a few scales have been developed in China including QOL Scale for Psoriasis Patients Treated with TCM by Zhou [29], the Psoriasis QOL Scale compiled with the connotation of Chinese medicine by Wang [30], the PQOLS (Psoriasis QOL Scale for Chinese Patients) compiled by Chen et al. [31]. However, they also have not been developed based on the modular approach, and most of them only used in traditional Chinese medicine [29, 30].

To solve these problems, we developed a QOL system called QLICD (Quality of Life Instruments for Chronic Diseases), which included a general module (QLICD-GM) that could be used for various diseases and some disease-specific modules [32]. At present, the latest version of the system was QLICD (V2.0), which contained 32 chronic disease-specific scales [33], including QLICD-CG [34], QLICD-PT [35], etc. The research mainly developed the specific module for patients with psoriasis, and then combined it with the general module that had been developed to form the Psoriasis Scale (QLICD-PS V2.0) in the System. This article aimed to report the development and validation of this scale.

## Methods

### Development of the QLICD-PS

#### General principles and steps of developing the QLICD-PS

**Fig. 1:**
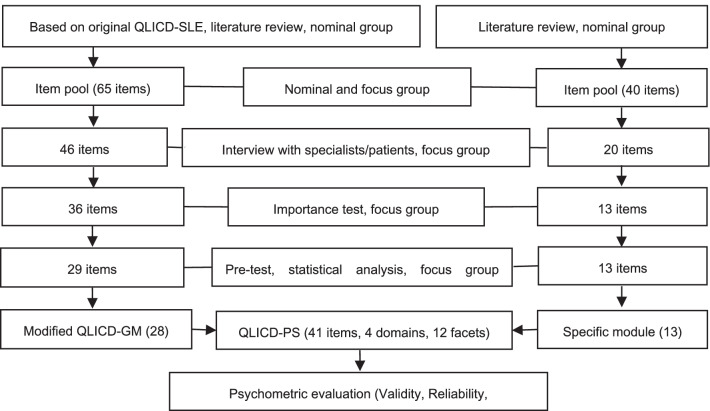
Steps towards development and validation procedure of QLICD-PS

The psoriasis disease-specific module was completed through the efforts of two independent groups. The nominal group consisted of 16 people, including 6 doctors, 2 nurses, 1 medical educator, and 7 teachers/researchers (2 QOL researcher, 2 psychologists, 2 sociologists, and 1 epidemiologist), which proposed the item pool using programmatic decision-making method. The focus group was composed of 10 experts, including 4 doctors, 1 medical educator and 5 teachers/researchers (2 QOL research scholars, 1 epidemiologist, 1 sociologist, and 1 psychologist), which proposed the conceptual framework using programmatic decision-making method and selects items proposed by the nominal group. In general, the nominal group was mainly responsible for reviewing the literature, referring to existed QOL scales for psoriasis patients and combining the clinical symptoms of psoriasis to propose items. The focus group was more specialized and refined, and was mainly responsible for screening, discussing and revising the items proposed by the nominal group. In the item selection process, both qualitative analysis methods such as group discussions, in-depth interviews as well as quantitative statistical methods such as variation analysis, correlation analysis, and factor analysis were used. Through a series of screening of the items, we had determined the specific module of the psoriasis scale, which were kept to 13 items (coded as PS1-PS13) and divided into 3 facets. It formed the QLICD-PS with the developed general module (QLICD-GM) (See Fig. 1 in detail).

#### Development of the QLICD-GM

The development of the QLICD-GM strictly followed the internationally recognized method of programmatic decision-making [32], and mainly included the following steps:(1) Establish a scale research team; (2) Define and decompose QOL measurement concepts to form a theoretical framework; (3) Propose a pool of candidate items; (4) Screen items to form a preliminary scale; (5) Screen pre-survey items to form a test scale; (6) Re-screen test surveys and items; (7) Evaluate the scale; (8) Form the formal scale. In the end, the QLICD-GM of 28 items was developed including 3 domains of physical function (9 items), mental function (11 items) and social function (8 items), which can be classified into 9 facets.

#### Creating the specific module of psoriasis

Using a similar procedure as described above, based on literature review, nominal group/focus group discussions, and patient interviews, we selected 20 items from the 40 item pool of the psoriasis-specific module. After pre-investigation and two stage screenings, the final module of 13 items (coded as PS1-PS13) was developed including 3 facets of specific symptoms (SPS), treatment side effects (TSE), and psychosocial impact of psoriasis (PSI) (Fig. 1).

### Validation of the QLICD-PS

#### Data collection

A combination of the general module QLICD-GM and the newly developed psoriasis specific module was used to form the QLICD-PS. It was used for on-site investigation and evaluation of patients with psoriasis. The survey was conducted in the Dermatology Department of the First Affiliated Hospital of Zhengzhou University in China. The research objects were psoriasis patients with certain reading comprehension ability and ability to fill out the questionnaire independently. The investigators (doctors, nurses and medical graduate students) explained the purpose and significance of the study to the patients, and obtained the informed consent of the patients who agreed to participate in the study. The research protocol and informed consent form were approved by the ethics committee of the survey institution.

Each patient (n = 122) completed the first questionnaire before receiving treatment as the first round of assessment. After 1–2 days of treatments, the respondents (n = 110) participated in the second round of assessment. After 1 week of treatments, a total of 122 patients participated in the third round of assessment for responsiveness. Due to the lack of a recognized gold standard, we used the Chinese version of the SF-36 to collect data so as to evaluate the criterion-related validity of QLICD-PS.

#### The scoring method of the QLICD-PS

Each item of QLICD-PS was scored in a five-level Likert scoring system, that is, none, a little, average, fairly and very. Forward entries were scored from 1 to 5, while reverse entries were scored from 5 to 1. By adding up the domain/facet item scores, we obtained the raw scores of facet and domain. The total score of the scale was the sum of the scores in all domains. For comparison, the following equation was used to linearly convert all domain scores into standardized scores (SS) between 0 and 100: SS = (RS-Min) × 100/R, where RS, Min, and R represented the original score, the lowest score and score range (Table 2).

**Table 2:** The Construct and scoring method of the quality of life instrument QLICD-PS (V2.0)

Domain/Facets	Number of items	Min	Max	Raw score	Standardized score
Physical domain (PHD)	9	9	45	BPF + IND + EAD	(RS-9) × 100/36
Basic physical functions (BPF)	4	4	20	GPH1 + GPH2 + GPH3 + GPH4	(RS-4) × 100/16
Independence (IND)	3	3	15	GPH6 + GPH7 + GPH8	(RS-3) × 100/12
Energy and discomfort (EAD)	2	2	10	GPH5 + GPH9	(RS-2) × 100/8
Psychological domain (PSD)	11	11	55	COG + EMO + WIP	(RS-11) × 100/44
Cognition (COG)	2	2	10	GPS1 + GPS2	(RS-2) × 100/8
Emotion (EMO)	7	7	35	GPS3 + GPS4 + GPS5 + GPS6 + GPS7 + GPS8 + GPS9	(RS-7) × 100/28
Will and personality (WIP)	2	2	10	GPS10 + GPS11	(RS-2) × 100/8
Social domain (SOD)	8	8	40	INC + SSS + SOR	(RS-8) × 100/32
Interpersonal communication (INC)	3	3	15	GSO1 + GSO2 + GSO3	(RS-3) × 100/12
Social support and security (SSS)	3	3	15	GSO4 + GSO5 + GSO6	(RS-3) × 100/12
Social role (SOR)	2	2	10	GSO7 + GSO8	(RS-2) × 100/8
Sub-total (QLICD-GM)	28	28	140	PHD + PSD + SOD	(RS-28) × 100/112
Specific domain (SPD)	13	13	65	SPS + TSE + PSI	(RS-14) × 100/52
Specific symptoms (SPS)	5	5	25	PS1 + PS5 + PS6 + PS7 + PS8	(RS-5) × 100/20
Treatment side effects (TSE)	3	3	15	PS9 + PS10 + PS11	(RS-3) × 100/12
Psychological impact (PSI)	5	5	25	PS2 + PS3 + PS4 + PS12 + PS13	(RS-5) × 100/20
Total (TOT)	41	41	205	PHD + PSD + SOD + SPD	(RS-41) × 100/164

Based on scores, we completed the assessment of QLICD-PS from the perspectives of reliability (internal consistence and test–retest reliability), validity (construct validity, content validity, and criterion-related validity) and responsiveness.

#### Internal consistency (reliability)

This study evaluated the reliability of the scale from two aspects: internal consistent reliability and test–retest reliability. Cronbach’s alpha coefficient is common practice in scale development to evaluate the internal consistency of reliability, with coefficient between 0.70 and 0.95 being regarded as evidence of sufficient internal consistency [36]. Sehunemann [37] proposed that the test–retest reliability between 0.73and 0.95 was adequate. To assess internal consistency, Cronbach’s alpha coefficient and test–retest reliability was calculated separately for each domain/facet.

#### Content validity

The content validity refers to whether the designed item/scale could represent the content or topic to be measured. Qualitative analysis including by discussions of the nominal group and the focus group was adopted to evaluate content validity.

#### Construct validity

Item-domain correlation analysis and exploratory factor analysis were used to evaluate the construct validity of the scale in this study. We performed Pearson’s correlation coefficient r among items and domains with 0.40 or greater as the threshold [8, 36, 38]. We performed Exploratory factor analysis based on the eigenvalues > 1 criterion to examine the coincidence between components extracted from data and theoretical construct of the instrument, and to display and confirm the construct validity clearly by Varimax rotation with factor loadings greater 0.50 as criterion [8, 38]. Multi-trait scaling analysis (Pearson’s correlation analysis in fact) [36] was employed to test item convergent validity and discriminant validity, with the following criteria: convergent validity is supported when an item-domain correlation is greater 0.40; and discriminant validity is revealed when item-domain correlation is higher than that with other domains.

#### Criterion-related validity

This study used the SF-36 as the Criterion to confirm criterion-related validity. The SF-36 includes eight subscales (domains): Physical Function (PF), Role-Physical (RP), Bodily Pain (BP), General Health (GH), Vitality (VT), Social Function (SF), Role-Emotional (RE) and Mental Health (MH). We calculated Pearson’s correlation coefficients between the similar domains of QLICD-PT and SF-36 to evaluate criterion-related validity.

#### Responsiveness

Responsiveness referred to the ability of the scale to detect small clinically important changes over time [39, 40]. This study mainly calculated the average scores of each domain/facet of the QLICD-PS at the first and third assessments (before and after treatments). The paired *t* test was used to evaluate responsiveness with calculating the standard responsiveness mean (SRM). The SRM was the ratio (absolute value) of the difference before and after treatment to the standard deviation of the difference. Husted [40] suggested that SRM above 0.8 indicated a very good responsiveness, SRM around 0.5 indicated a moderate responsiveness, and SRM around 0.2 indicated a low responsiveness.

## Results

### Socio-demographic characteristics of the sample

The age of 122 patients with psoriasis ranged from 21 to 57 years old, with an average age of 39.73 years. 78 cases (63.9%) were male and 117 cases (95.9%) were Han nationality. Most participants were married (84 cases, 68.9%) and 29 cases (23.8%) were widowed. In terms of educational level, 14 participants were graduated from primary school (11.5%), 43 graduated from secondary school (35.2%), 47 graduated from high school or technical secondary school (38.5%), and 18 graduated from college or university (14.8%). Among them, 45 were farmers (36.9), 34 were workers (27.9%), 3 were teachers (2.5%), and 5 were cadres (4.1%). Most of the forms of medical insurance were cooperative medical care (n = 57, 46.7%).

### Internal consistency (reliability)

The Cronbach's α of each domain of the QLICD-PS was between 0.59 and 0.87. The Cronbach's α of the specific module, the general module and the total scale were 0.78, 0.87 and 0.74, respectively. The split-half reliability of each domain of the QLICD-PS was between 0.73 and 0.91. The split-half reliability of the specific module, the general module and the total scale were 0.81, 0.91 and 0.81, respectively. Both of these results showed good internal consistency reliability (see Table 3 in detail).

**Table 3:** Internal consistent of the quality of life instrument QLICD-PS (n = 122)

Domain/Facets	Number of Entries	Alpha coefficient	Split-half reliability
Physical domain (PHD)	9	0.801	0.844
Basic physical functions (BPF)	4	0.585	0.571
Independence (IND)	3	0.905	0.872
Energy and discomfort (EAD)	2	*	*
Psychological domain (PSD)	11	0.809	0.862
Cognition (COG)	2	*	*
Emotion (EMO)	7	0.768	0.821
Will and personality (WIP)	2	*	*
Social domain (SOD)	8	0.586	0.728
Interpersonal Communication (INC)	3	0.448	0.388
Social support and security (SSS)	3	0.355	0.568
Social role (SOR)	2	*	*
Specific domain (SPD)	13	0.784	0.813
Specific symptoms (SPS)	5	0.517	0.586
Treatment side effects (TSE)	3	0.706	0.725
Psychological impact (PSI)	5	0.815	0.813
General module (GM)	28	0.869	0.907
Total (TOT)	41	0.735	0.811

^*^Not suitable for calculation

### Test–retest reliability

Correlation analysis and paired *t*-tests were performed on various domains/facets, and the results showed that the differences of physical function, psychological function, social function and the general module as well as the total scale in the first and second evaluations were not statistically significant (*P* > 0.05). At the same time, the correlation analysis results showed that there was a significant correlation in each domain/facet (*P* < 0.001), with correlation coefficients being between 0.84 and 0.94. The test–retest reliability coefficient of the specific module and the total scale was 0.85 and 0.94, respectively. These results indicated that the QLICD-PS scale had good test–retest reliability (See Table 4 in detail).

**Table 4:** Test–retest reliability of the quality of life instrument QLICD-PS (n = 122)

Domain/Facets	First measurement		Second measurement		Paired *t* test		Correlation	
	Average	Standard deviation	Average	Standard deviation	*t*	*P*	*r*	*P*
PHD	65.51	17.28	66.41	16.53	− 1.36	**0.177**	**0.92**	< 0.001
BPF	62.67	16.79	62.95	16.52	− .288	0.774	0.81	< 0.001
IND	78.56	27.03	78.48	26.46	0.070	0.944	0.91	< 0.001
EAD	51.59	25.35	55.23	24.62	− 2.725	0.008	0.84	< 0.001
PSD	55.00	15.81	54.83	17.11	0.182	**0.856**	**0.84**	< 0.001
COG	63.30	19.55	62.39	19.85	0.807	0.422	0.82	< 0.001
EMO	52.18	17.80	68.99	18.23	− 7.801	0.000	0.21	< 0.05
WIP	56.59	22.19	57.84	23.64	− 1.225	0.223	0.89	< 0.001
SOD	67.27	12.90	67.95	13.58	− 1.138	**0.258**	**0.89**	< 0.001
INC	72.42	15.00	73.48	15.02	− 1.136	0.191	0.84	< 0.001
SSS	66.66	17.27	67.80	16.42	− 1.680	0.096	0.91	< 0.001
SOR	60.45	23.00	59.89	23.77	0.432	0.667	0.83	< 0.001
SPD	59.55	14.82	57.92	17.61	1.811	**0.073**	**0.85**	< 0.001
SPS	69.32	15.60	63.32	19.66	4.352	0.000	0.69	< 0.001
TSE PSI	78.56 42.00	27.03 24.00	78.48 43.45	26.45 25.30	0.070 − 1.360	0.944 0.177	0.91 0.90	< 0.001 < 0.001
GM	61.88	12.35	62.31	12.99	− 1.019	**0.311**	**0.94**	< 0.001
TOT	61.14	11.30	60.91	12.91	0.524	**0.601**	**0.94**	< 0.001

Bold indicates more important values

### Content validity

After repeated discussions by the nominal group and the focus group, the QLICD-PS was compiled according to a strict procedural method, with items including all the dimensions and assessment content. The scale included physical, psychological, social and clinical symptoms of patients with psoriasis, side effects of treatment, and special psychological characteristics of the disease. It fully reflected the connotation of the QOL of patients with psoriasis, indicating good content validity.

### Construct validity

Correlation analyses displayed that there were sufficiently associations between items and their own domains/facets (most correlation coefficients are greater than 0.5), but weak associations between items across domains/facets and between domains/facets (Table 5). For example, correlation coefficients between items of GPH1–GPH9 (in bold) are greater than those across domains.

**Table 5:** Correlation coefficients r among items and domains/facets of QLICD-PS (n = 122)

Items	Physical domain	Psychological domain	Social domain	Specific module
GPH1	0.484**	0.259**	0.383**	− 0.095
GPH2	0.614**	0.330**	0.369**	− 0.179*
GPH3	0.361**	0.067	0.180*	− 0.222*
GPH4	0.495**	0.325**	0.279**	− 0.137
GPH5	0.665**	0.280**	0.206*	− 0.299**
GPH6	0.753**	0.234**	0.320**	− 0.252**
GPH7	0.828**	0.254**	0.402**	− 0.451**
GPH8	0.771**	0.248**	0.346**	− 0.196*
GPH9	0.600**	0.248**	0.190*	− 0.263**
GPS1	0.555**	0.613**	0.618**	− 0.262**
GPS2	0.080	**0.160**	− 0.033	0.028
GPS3	0.312**	0.292**	0.278**	− 0.026
GPS4	0.138	0.474**	0.098	− 0.040
GPS5	0.230*	0.512**	0.415**	− 0.407**
GPS6	0.058	0.662**	0.453**	− 0.307**
GPS7	0.151	0.789**	0.456**	− 0.333**
GPS8	0.155	0.823**	0.496**	− 0.291**
GPS9	0.160	0.740**	0.448**	− 0.369**
GPS10	0.344**	0.645**	0.638**	− 0.095
GPS11	0.354**	0.729**	0.368**	− 0.305**
GSO1	0.436**	0.578**	0.687**	− 0.315**
GSO2	− 0.031	0.145	**0.157**	0.273**
GSO3	0.194*	0.200*	0.429**	0.088
GSO4	0.093	0.349**	0.446**	0.291**
GSO5	0.156	0.260**	0.594**	− 0.080
GSO6	0.229*	0.288**	0.471**	− 0.498**
GSO7	0.286**	0.365**	0.549**	− 0.537**
GSO8	0.434**	0.427**	0.643**	− 0.270**
PS1	− 0.107	− 0.243**	− 0.057	0.473**
PS2	− 0.378**	− 0.294**	− 0.288**	0.623**
PS3	− 0.367**	− 0.178*	− 0.280**	0.604**
PS4	− 0.071	0.172	− 0.015	0.366**
PS5	− 0.073	0.287**	0.121	0.172
PS6	− 0.165	− 0.053	− 0.066	0.389**
PS7	− 0.177	− 0.090	− 0.175	0.586**
PS8	− 0.280**	− 0.260**	− 0.220*	0.611**
PS9	− 0.121	− 0.493**	− 0.375*	0.588**
PS10	− 0.172	− 0.323**	− 0.284*	0.614**
PS11	− 0.316**	− 0.469**	− 0.380**	0.699**
PS12	− 0.188*	− 0.257**	− 0.134	0.522**
PS13	− 0.286**	− 0.318**	− 0.298**	0.602**

Bold indicates more important values

^**^P < 0.01, *P < 0.05

**Fig. 2:**
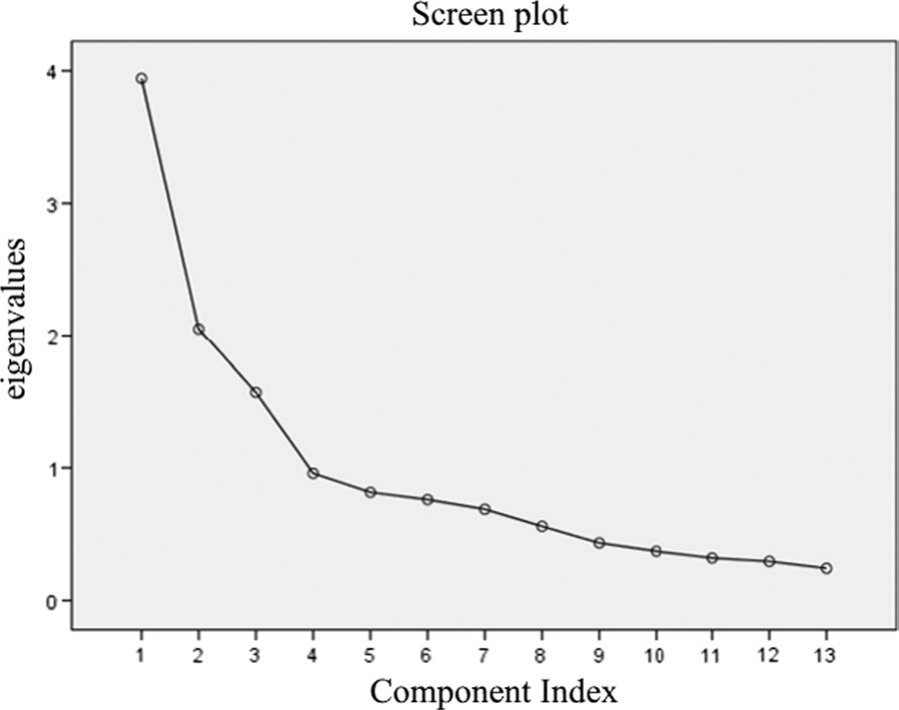
Screen plot of eigenvalues after pca

The Kaiser–Meyer–Olkin value of the specific module was 0.747, and Bartlett’s Tests of Sphericity was statistically significant (*P* < 0.005), supporting suitability of factor analysis. The factor analysis extracted 3 principal components from the 13 items of the specific module with the cumulative variance of 58.157%, reflecting 3 facets of this module (Table 6). The contribution rates of the first, second and third principal components were 24.793%, 17.690% and 15.674% respectively (Fig. 2).

**Table 6:** Principal components and factor loadings of the specific module of QLICD-PS (n = 122)

Items	Brief description	Principal components and variance contribution (%)		
		P1(24.793%)	P2(17.690%)	P3(15.674%)
PS1	Itchy skin	0.197	− 0.075	**0.838**
PS2	Skin desquamation	0.417	0.164	**0.575**
PS3	Swelling and pain in the skin lesion	0.234	**0.631**	0.209
PS4	Pustules on the skin	0.158	**0.704**	− 0.389
PS5	Pain in limbs and joints	− 0.295	**0.616**	− 0.045
PS6	Skin itching or burning after using topical medicine	− 0.141	0.372	**0.694**
PS7	Skin discomfort after using topical hormone drugs	0.130	**0.645**	0.390
PS8	Dry mouth or throat after taking the medicine	0.253	**0.608**	0.271
PS9	Worried that psoriasis could be inherited	**0.711**	0.120	0.045
PS10	Impact on the appearance	**0.720**	0.163	0.037
PS11	Being alienated or discriminated	**0.835**	− 0.006	0.258
PS12	Impact on love and marriage	**0.644**	− 0.068	0.159
PS13	Avoid going to public places	**0.779**	0.152	− 0.065

Bold indicates more important values

By using the Varimax rotation method, it can be seen clearly that the 3 principal components reflected 3 different facets of the specific module. The first component represented the facet of Psychosocial impact with higher factor loadings on PS9 (0.71), PS10 (0.72), PS11 (0.84), PS12 (0.64) and PS13 (0.78); the second component reflected mainly treatment side effects with higher factor loadings on PS3 (0.63), PS4(0.70), PS5(0.61), PS7(0.65) and PS8(0.61); the third component mainly represented the specific symptoms with higher factor loadings on PS1(0.83) and PS2(0.58).

The above analysis results confirmed the theoretical construct, showing good construct validity.

### Criterion-related validity

Correlation coefficients among the domain scores of the QLICD-PS and SF-36 were expressed in the Table 7, showing that the correlations between the same and similar domains are generally greater than those between different and non-similar domains. For example, the coefficient between the physical of QLICD-PS and physical function of SF-36 was 0.64, greater than any other coefficients in this row. It was considered that the criterion-related validity of the QLICD-PS was good.

**Table 7:** Correlation coefficients among domains scores of QLICD-PS and SF-36 (n = 122)

SF-36	QLICD-PS			
	Physical	Psychological	Social	Specific module
Physical function	0.64**	0.29**	0.43**	− 0.25**
Role-physical	0.44**	0.17	0.41**	− 0.17
Body pain	− 0.58**	− 0.30**	− 0.36**	0.48**
General health	− 0.20*	− 0.41**	− 0.41**	0.31**
Vitality	− 0.03	− 0.11	− 0.12	**0.16**
Social function	0.21*	0.10	0.20**	− 0.10
Role-emotional	0.30**	0.15	0.22*	0.09
Mental health	0.091	0.12	**0.15**	**0.15**

Bold indicates more important values

All coefficients have statistical significance **P < 0.01, *P < 0.05

### Responsiveness

The results in Table 8 showed that all domains of the scale were statistically significant (*P* < 0.05). SRM in various domains ranged from 0.12 to 0.58, with that of the specific module and the total scale being 0.58 and 0.51, respectively. It could be seen that the responsiveness of the QLICD-PS was moderate (Table 8).

**Table 8:** Responsiveness of the quality of life instrument QLICD-PS (n = 122)

Domain/Facets	Before treatment	After treatment	Paired *t* test		*SRM*
	$\bar{x}\pm s$	$\bar{x}\pm s$	*T*	*P*	
Physical domain (PHD)	**64.85 ± 17.66**	**73.48 ± 12.61**	− 9.327	** < 0.001**	**0.49**
Basic physical functions (BPF)	62.30 ± 16.37	70.08 ± 13.06	− 8.038	< **0.001**	0.48
Independence (IND)	77.87 ± 28.68	82.51 ± 23.14	− 3.446	0.001	0.16
Energy and discomfort (EAD)	50.41 ± 25.76	66.70 ± 18.15	8.641	< 0.001	0.63
Psychological domain (PSD)	**54.88 ± 16.33**	**54.88 ± 16.33**	− 4.502	< **0.001**	**0.26**
Cognition (COG)	62.10 ± 19.87	63.93 ± 20.08	− 1.530	0.129	0.09
Emotion (EMO)	52.28 ± 18.16	57.55 ± 15.46	− 4.478	< 0.001	0.29
Will and personality (WIP)	56.76 ± 22.85	60.04 ± 20.75	− 2.523	0.013	0.14
Social domain (SOD)	**67.14 ± 13.50**	**68.70 ± 13.60**	− 2.197	**0.030**	**0.12**
Interpersonal communication (INC)	71.80 ± 15.94	75.61 ± 14.34	− 4.433	< 0.001	0.24
Social support and security (SSS)	67.00 ± 17.42	68.03 ± 16.78	− 1.018	0.311	0.06
Social role (SOR)	60.35 ± 23.71	59.32 ± 23.10	0.844	0.400	0.04
Specific domain (SPD)	**56.81 ± 16.45**	**66.27 ± 13.65**	− 8.032	< **0.001**	**0.58**
Specific symptoms (SPS)	60.16 ± 18.40	78.98 ± 13.79	− 11.294	< 0.001	1.08
Treatment side effects (TSE)	72.06 ± 21.64	74.60 ± 18.83	− 1.730	0.086	0.12
Psychological impact (PSI)	44.30 ± 24.73	48.57 ± 25.46	− 3.016	0.003	0.17
General module (GM)	61.59 ± 13.03	66.49 ± 11.21	− 8.167	< **0.001**	**0.38**
Total (TOT)	153.93 ± 31.35	170.20 ± 27.07	− 10.504	< 0.001	**0.51**

Bold indicates more important values

## Discussions

### Thoughts and characteristics of scale development

Based on the first version of QLICD-GM, the second version of QLICD-GM has made several improvements to increase the comprehensibility and accessibility [35]. By combining the improved general module QLICD-GM and the developed specific module for psoriasis, we developed a new QOL assessment scale for patients with psoriasis. The development process screened and modified the proposed item pool according to a rigorous procedural method, with 13 items being remained to form the final specific module. This modular approach unifies all disease-specific instruments of QLICDs under the same general module. Therefore, we can use QLICD-GM to capture general QOL in patients with different diseases, and then employ disease-specific modules such as QLICD-PT for pulmonary tuberculosis and QLICD-CG for Chronic Gastritis to catch the aspects of QOL that differentiate the different diseases. Therefore, the QLICD-PS is different from other QOL instruments for psoriasis.

### Evaluation of QLICD-PS

Generally speaking, a practical QOL should be verified in at least three aspects: reliability, validity and responsiveness [32, 35]. In this study, the internal consistent reliability, the split-half reliability and the test–retest reliability were used to evaluate reliability of the QLICD-PS, with the results showing good reliability. Correlation analyses, multi-trait scaling analysis and factor analysis were employed to display validity, with the results confirming good validity on the whole.

The responsiveness results of the scale in this study showed that SRM ranged from 0.12 to 0.58, among which the SRM in the domains of mental function and social function were low (0.27 and 0.12 respectively). It might be because psoriasis was a chronic itchy skin disease, and its recurring characteristics had a profound impact on the QOL of patients, especially in terms of psychological and social functions which were difficult to be improved during a short hospital stay. It implies that psychological and social functions were not sensitive to short-term treatments. According to the evaluation criteria of responsiveness, SRM of the specific module and the total scale (0.51 and 0.54) could be rated as moderate responsiveness. Most probably, psoriasis was a chronic skin disease and the patient's hospital stay was short, the specific module was not expected to change significantly before and after treatments in a short period of time.

In China, although some scales were developed based on Chinese culture and demonstrated acceptable psychometric properties [29–31], there were still some shortcomings. Firstly, these scales were not systematically developed by the modular approach (combination of the general module and specific modules), and thus cannot be used to compare different diseases. Second, these scales lacked a unified standard, and the measurement method needed to be improved. Moreover, they did not involve the impact of drug side effects on patients enough, and most of them only used in traditional Chinese medicine [29, 30]. The PQOLS can be used in patients treated in Western medicine, but only has 4 items on symptoms of the disease and no item on side effects of the drug [31]. Therefore, it was necessary to develop a scientific, reasonable, reliable and suitable QOL measurement scale for Chinese psoriasis patients. In this study, a combination of the general module of recognized and well-developed system of QOL instruments for chronic diseases (QLICD) with the newly developed psoriasis specific module was used to form QLICD-PS. Compared with other scales in China, the QLICD-PS not only has good validity, reliability and responsiveness, but also can compare QOL across diseases by the general module, demonstrating both generic and specific properties. Moreover, it has a significant advantage that it consists of a moderate number of items with a clear hierarchical structure (items → facets → domains → overall) so that analysis of scores can be carried out not only at the domain and the overall levels but also at the different facet levels (12 facets in all) to detect changes in detail.

### Strengths and limitations

Using the method of combining the general module of chronic diseases and the specific modules of diseases, the QOL assessment scale for psoriasis patients with Chinese cultural background was developed. Of course, this study was also subject to various restrictions. First, the sample size of the study was not very large for psoriasis is a skin disease with seasonal and regional characteristics, and the acquisition of samples was restricted by many unchangeable conditions such as seasons and regions. Furthermore, patients with psoriasis participated were limited to individuals who were able to read and understand the questionnaire in Chinese, the level of cultural proficiency should be carefully evaluated when translate QLICD-PS into other languages.

## Conclusions

The QLICD-PS was developed under the Chinese cultural background by combining the recognized general module of chronic diseases and the specific module of psoriasis with several advantages. The QLICD-PS had good validity, reliability and responsiveness, and can be used to measure the QOL of Chinese patients with psoriasis.

## Data Availability

Please contact the authors for data requests.
